# The association between outcome-based quality indicators for intensive care units

**DOI:** 10.1371/journal.pone.0198522

**Published:** 2018-06-13

**Authors:** Ilona W. M. Verburg, Evert de Jonge, Niels Peek, Nicolette F. de Keizer

**Affiliations:** 1 Academic Medical Center, University of Amsterdam, Department: Medical Informatics, Amsterdam Public Health research institute, Amsterdam, The Netherlands; 2 Department of Intensive Care, Leiden University Medical Center, Leiden, the Netherlands; 3 Division of Informatics, Imaging, and Data Science, University of Manchester, Manchester, United Kingdom; University of Colorado Denver, UNITED STATES

## Abstract

**Purpose:**

To assess and improve the effectiveness of ICU care, in-hospital mortality rates are often used as principal quality indicator for benchmarking purposes. Two other often used, easily quantifiable, quality indicators to assess the efficiency of ICU care are based on readmission to the ICU and ICU length of stay. Our aim was to examine whether there is an association between case-mix adjusted outcome-based quality indicators in the general ICU population as well as within specific subgroups.

**Materials and methods:**

We included patients admitted in 2015 of all Dutch ICUs. We derived the standardized in-hospital mortality ratio (SMR); the standardized readmission ratio (SRR); and the standardized length of stay ratio (SLOSR). We expressed association through Pearson’s correlation coefficients.

**Results:**

The SMR ranged from 0.6 to 1.5; the SRR ranged from 0.7 to 2.1; and the SLOSR ranged from 0.7 to 1.3. For the total ICU population we found no significant associations. We found a positive, non-significant, association between SMR and SLOSR for admissions with low-mortality risk, (r = 0.25; p = 0.024), and a negative association between these indicators for admissions with high-mortality risk (r = -0.49; p<0.001).

**Conclusion:**

Overall, we found no association at ICU population level. Differential associations were found between performance on mortality and length of stay within different risk strata. We recommend users of quality information to take these three outcome indicators into account when benchmarking ICUs as they capture different aspects of ICU performance. Furthermore, we suggest to report quality indicators for patient subgroups.

## Introduction

In recent years attention to quality of healthcare as expressed by quality indicators has increased [1, 2]. Treatment at the intensive care unit (ICU) is very complex and delivered in a highly technical and labor-intensive environment. Along with these developments the cost of intensive care has increased substantially resulting in a high proportion of the health care expenditure accountable to ICUs [3]. This all makes ICUs a particularly interesting part of the hospital to assess and improve performance.

Within the ICU setting several outcome, structure and process quality indicators are presented [4, 5]. This study focusses on three outcome indicators for ICU care. To assess and improve the effectiveness of ICU care, in-hospital mortality rates are often used as principal quality indicator. Two other often used, easily quantifiable, quality indicators to assess the efficiency of ICU care are based on readmission to the ICU and ICU length of stay. ICU length of stay is highly associated with the costs of ICU care and an easily quantifiable outcome indicator for efficiency of ICU care [6, 7]. ICU discharge is often based on subjective criteria and discharge policy could be influenced by the availability of bed and staff resources [8–10].

Premature discharge may lead to an increase in patients readmitted to the ICU or higher post-ICU mortality rates [11–15]. Improving discharge decision making may reduce resource consumption, prevent readmissions to the ICU, shorten ICU length of stay, and hence reduce costs [16].

Several studies have shown patient level associations. Patients having an ICU readmission are more severely ill, show longer ICU length of stay and higher in-hospital mortality rates compared to patients not having an ICU readmission [17, 18]. Furthermore, ICU length of stay differs for survivors compared to non-survivors [19, 20]. This study focuses on the association between these quality indicators on unit-level in the context of benchmarking the performance of ICUs.

To get a more complete picture of these quality indicators when comparing ICUs, presenting several quality indicators simultaneously may have added value. Quality indicators could be positively correlated, that is, ICUs performing well on one of the quality indicators may also perform well on other quality indicators. This means that the quality indicator does not only provide information about the related process, on efficiency or effectiveness, but it also provides information about quality in general. Quality indicators could also be negatively correlated, meaning that performing well on one of the quality indicators often comes at the expense of performance on the others. It could be the case that these quality indicators are not correlated because the quality indicators capture independent aspects of performance. Finally, it is relevant to know whether ICUs perform similarly across their entire patient population or differently in specific subgroups.

The aim of this study was to examine whether there is an association between unit-level, case-mix adjusted quality indicators based on in-hospital mortality; readmission to the ICU; and ICU length of stay in the general Dutch ICU population as well as within specific subgroups of ICU patients.

## Materials and methods

### The Dutch National Intensive Care Evaluation registry

The NICE registry has been active since 1996 and, in 2015, all 83 Dutch ICUs delivered data to the registry. The NICE registry [21] collects demographical, physiological and diagnostic data from the first 24 hours of all patients admitted to participating ICUs, including all variables used in the Acute Physiology and Chronic Health Evaluation (APACHE) IV model to predict probabilities of in-hospital mortality [22]. Collected data is checked by registry staff for internal consistency, by performing onsite data quality audits and training local data collectors [23].

The data collected by the registry are officially registered and collected in accordance with the Dutch Personal Data Protection Act. The medical ethics committee of the Academic Medical Center waived the need for medical ethics for this study (registration number W17_162).

### Study data

**Fig 1:**
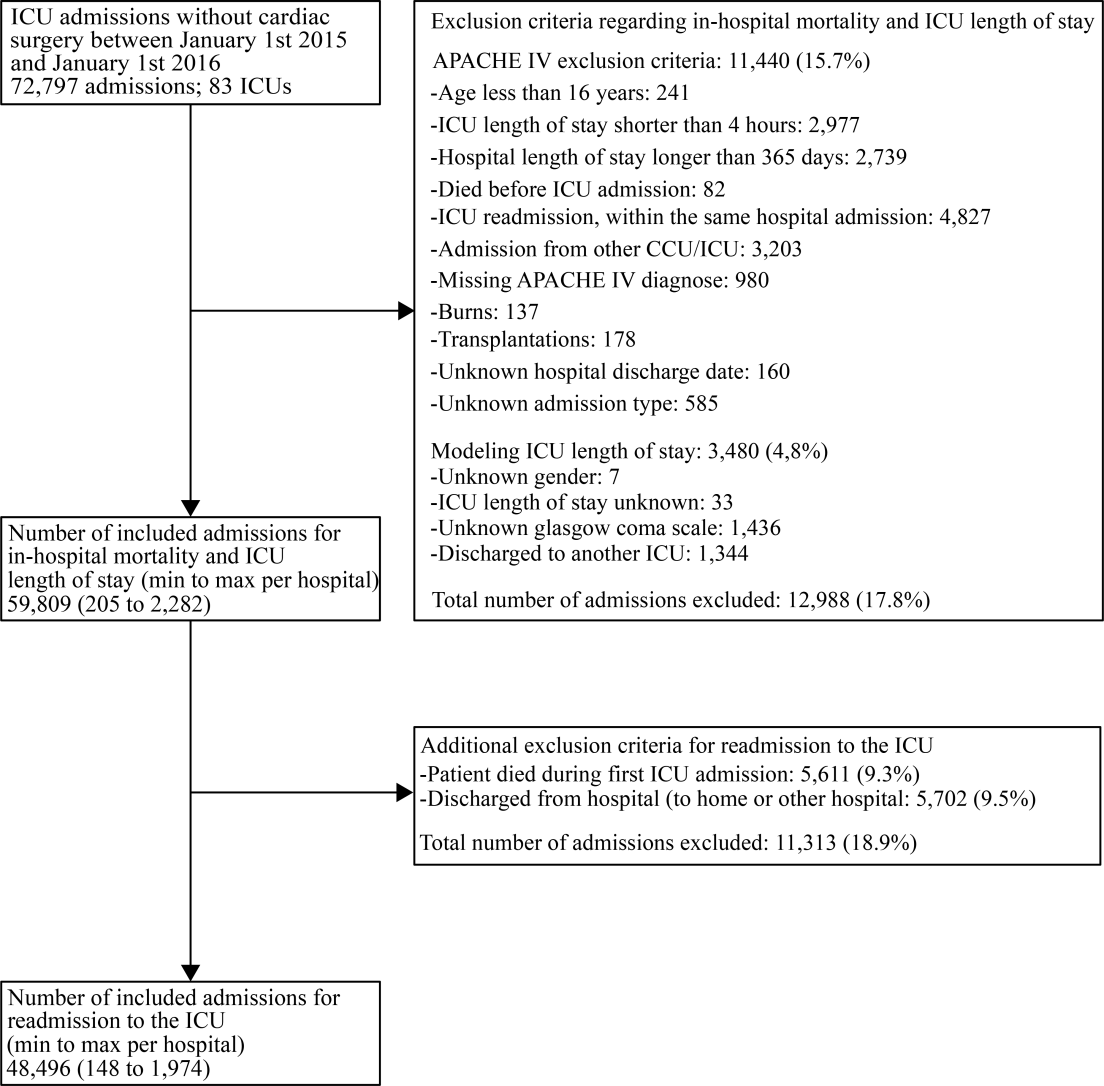
Flowchart of admissions included for the quality indicators for in-hospital mortality, readmission to the ICU and ICU length of stay.

We included consecutive ICU admissions with initial ICU admission between January 1^st^ 2015 and January 1^st^ 2016. Fig 1 presents a flow chart of patient inclusion. We excluded cardiac surgery patients, as patients admitted for cardiac surgery in general have a short, generally fixed, ICU length of stay. We applied the APACHE IV exclusion criteria [22]. Since patient data are anonymized, we can only match different ICU admissions of a patient within the same hospitalization period. For this reason we excluded patients who were discharged to another ICU, as their observed ICU length of stay was truncated, and we excluded patients with missing values for model covariates.

A readmission to the ICU was defined as a second ICU admission within 48 hours of ICU discharge from the initial ICU admission, in the same hospital within the same hospital stay. A time period of 48 hours was chosen, since readmissions within this period have a stronger relationship with ICU interventions, such as mechanical ventilation, and discharge circumstances, than later readmissions [24, 25]. When considering readmissions to the ICU we excluded patients who were not at risk for readmission to the ICU during the same hospitalization period: patients who died during their initial ICU admission and patients who were discharged from the ICU to a location outside the hospital (e.g. to home, nursery home or another hospital) [24].

The NICE board has established subgroups of ICU admissions, of which the NICE registration publishes quality indicators [26]. Associations were examined for the entire cohort and for these subgroups: medical admission, urgent surgery or elective surgery; groups based on severity of illness defined using the calibrated APACHE IV probability of mortality (low: smaller than 0.3, medium: larger or equal to 0.3 and smaller than 0.7, or high: larger or equal to 0.7); and several homogeneous diagnostic subgroups, i.e. patients admitted for community acquired pneumonia (CAP), sepsis, or out of hospital cardiac arrest (OHCA). The precise definitions of all subgroups are presented in S1 Table.

### Quality indicators

Quality indicator scores may be influenced by differences in patient characteristics. Therefore, we performed case-mix correction to adjust for these differences. For case-mix correction we used patient characteristics available at admission time or within the first 24 hours of ICU admission. For all outcome measures case-mix correction models and model performances are presented in S1 File. Furthermore, the included ICUs differ in size. For subgroups of ICU patients with a small number of admissions, the estimators of quality indicators scores for some small ICUs would be less reliable than for ICUs with a larger number of admissions. For this reason we adjusted the scores for reliability using empirical Bayes estimators, by effectively shrinking unreliable scores (i.e. of small ICUs) towards 1 [27, 28]. The empirical Bayes estimators were implemented by fitting mixed effects regression models with random intercept for ICU [27, 28]. To avoid that subgroup analyses with small numbers of admissions would lead to non-convergence of the mixed effects regression analyses, we applied a two-step approach. First we fitted fixed effect models to calculate predicted values for each of the three outcome measures at patient level. These models were used for case-mix correction in the entire cohort and in subgroups. In a second step, the (log odds of the) predicted outcome was included as fixed covariate in mixed effect models for each of the three outcome measures and each subgroup.

Mortality: As quality indicator for in-hospital mortality after or during ICU admission we used the standardized mortality ratio (SMR). To estimate the reliability adjusted SMR, we first recalibrated the APACHE IV probability of mortality [29] to our dataset using logistic regression analysis. Secondly, for each subgroup we performed logistic regression with the log-odds of the probability of mortality as fixed covariates and ICU as random intercept. To estimate the reliability adjusted risk adjusted rates we subsequently added the log-odds of the overall mortality rate to the random intercept per ICU and applied the inverse logit of the result in order to calculate the reliability adjusted risk adjusted rates for each ICU. We divided the risk adjusted rates by the overall mortality rate to obtain the reliability adjusted SMR values.

Readmission to the ICU: As quality indicator for readmission to the ICU we used the standardized readmission ratio (SRR). To estimate the reliability adjusted SRR, we first estimated the expected probability of readmission by developing a logistic regression model on the entire cohort, in a similar way as described in a previous study [24]. Secondly, we estimated the SRR using random intercepts per ICU as is done for the SMR.

ICU length of stay: As quality indicator for ICU length of stay we used the standardized length of stay ratio (SLOSR). To estimate the reliability adjusted SLOSR, we first estimated the expected ICU length of stay, in fractional days, using ordinary least square regression with a log-link function, as we did in a previous study [19]. Secondly, we applied ordinary least square regression with the expected ICU length of stay as fixed covariate and ICU as random intercept. We added the random intercept per ICU to the average length of stay for each subgroup to obtain the reliability adjusted average ICU length of stay and divided this value by the average length of stay for the subgroup to get the reliability adjusted SLOSR.

### Statistical analysis

The overall performance for the models for in-hospital mortality and readmission to the ICU was assessed by the scaled Brier score [30] and the discriminative ability by the concordance index (C-statistic). The overall performance of the model for ICU length of stay was assessed by squared Pearson correlation coefficient (R^2^). Model calibration was assessed by creating 50 equally-sized subgroups of predicted values.

To present associations between quality indicators, we plotted pairs of quality indicator values (measured at unit level) against each other. We examined the association between pairs of quality indicator values by Pearson’s correlation coefficients and calculated corresponding p-values using the Student’s t-distribution. Because small numbers of patients per ICU may result in unreliable estimates of quality indicator values, we also performed a sensitivity analysis using Spearman’s rank correlation coefficient which is less sensitive to outliers. We considered p-values smaller than 0.01 as statistically significant.

We performed all analyses using the R statistical software, version 3.3.2 [31]. We used linear models (lm) and general linear models (GLM) for regression analysis [32] and the 'splines' package for calculating restricted cubic splines [33]. We used the lme4 package [34] to fit linear mixed-effects models (LMM) and generalized linear mixed-fixed models (GLMM) by respectively the functions 'lmer' and 'glmer' and estimated the random effects by the function 'ranef'. The 'cor.test' function was used to examine Pearson’s and Spearman’s correlation coefficients and corresponding p-values [35].

## Results

### Study data and quality indicators

In total 72,797 patients were admitted to 83 Dutch ICUs, for other reasons than admission after cardiac surgery. S2 Table presents the number of admissions per subgroup. For in-hospital mortality and ICU length of stay as outcome measure, after applying the exclusion criteria 59,809 (82.2%) patients, ranging from 205 to 2,282 patients per ICU were included. From these patients 48,496 (81%) were included for readmission as outcome measure, see Fig 1.

Table 1 presents crude percentages of the outcomes and case-mix adjusted quality indicators, for the entire cohort and each subgroup. Crude in-hospital mortality was 14.3% ranging from 2.3% to 26.4% and the recalibrated SMR ranged from 0.6 to 1.7. The crude percentage of ICU readmissions was 2.4% ranging from 0.0% to 5.5% and the SRR ranged from 0.7 to 2.1. The overall mean ICU length of stay was 3.3 ranging from 1.7 to 6.1 and the SLOSR ranged from 0.7 to 1.3. Associations based on the Pearson correlation coefficient were r = 0.40 (p<0.001) between the crude in-hospital mortality rate and SMR; r = 0.89 (p<0.001) between the crude readmission rate and SRR; and r = 0.48 (p<0.001) between the mean ICU length of stay and SLOSR.

**Table 1:** Values for the crude outcomes and for the quality indicators standardized mortality ratio (SMR); standardized readmission ratio (SRR) and standardized ICU length of stay ratio (SLOSR). Overall values are shown with minimum and maximum ranges over the ICUs.

	In-hospital mortality		Readmission to the ICU within 48 hours after ICU discharge		ICU length of stay	
Patient subgroup	Crude overall in-hospital mortality rate (min to max) (%)	Overall in-hospital SMR (min to max)	Crude ICU readmission rate (min to max) (%)	Overall SRR (min to max)	Overall median ICU length of stay (min to max)	SLOSR (min to max)
All ICU admissions	14.3 (2.3 to 26.4)	(0.6 to 1.5)	2.4 (0.0 to 5.5)	(0.7 to 2.1)	1.2 (0.9 to 2.8)	(0.7 to 1.3)
CAP	16.2 (0 to 100)	(0.7 to 1.6)	3.1 (0.0 to 33.3)	(0.9 to 1.2)	1.8 (0.4 to 20.8)	(0.7 to 1.4)
Sepsis	27.2 (0 to 55.3)	(0.8 to 1.3)	2.6 (0.0 to 14.3)	(0.8 to 1.4)	2.3 (0.7 to 11.4)	(0.8 to 1.3)
OHCA	48.7 (0 to 100)	(0.8 to 1.2)	2.9 (0.0 to 100)	(0.0 to 2.0)	3.0 (0.3 to 17.4)	(0.8 to 1.2)
*Admission type*						
Medical	19.5 (11.1 to 31.0)	(0.7 to 1.3)	2.6 (0.0 to 9.3)	(0.6 to 2.2)	1.8 (1.0 to 2.9)	(0.7 to 1.3)
Urgent Surgery	15.8 (2.3 to 30.4)	(0.6 to 1.4)	2.9 (0.0 to 11.5)	(0.8 to 2.1)	1.7 (0.8 to 4.7)	(0.8 to 1.2)
Elective surgery	3.3 (0.0 to 10.5)	(0.6 to 1.6)	2.0 (0.0 to 8.8)	(0.9 to 1.3)	0.9 (0.8 to 2.9)	(0.4 to 2.3)
*Probability of mortality*						
<0.3	6.6 (1.6 to 11.7)	(0.7 to 1.5)	2.3 (0.0 to 4.6)	(0.8 to 1.9)	1.0 (0.8 to 2.8)	(0.6 to 1.4)
≥0.3 and <0.7	47.7 (23.1 to 73.3)	(0.7 to 1.2)	3.9 (0.0 to 33.3)	(0.6 to 2.9)	3.5 (1.6 to 6.2)	(0.8 to 1.2)
≥0.7	78.4 (33.3 to 100)	(0.5 to 1.7)	3.2 (0.0 to 100)	(0.0 to 1.0)	2.2 (0.6 to 9.2)	(0.7 to 1.7)

SMR = standardized mortality ratio; SRR = standardized readmission ratio; SLOSR = standardized length of stay ratio; APACHE IV = Acute Physiology and Chronic Health Evaluation IV [22]; CAP = Community Acquired Pneumonia; OHCA = Out of Hospital Cardiac Arrest.

## Statistical analysis

**Fig 2:**
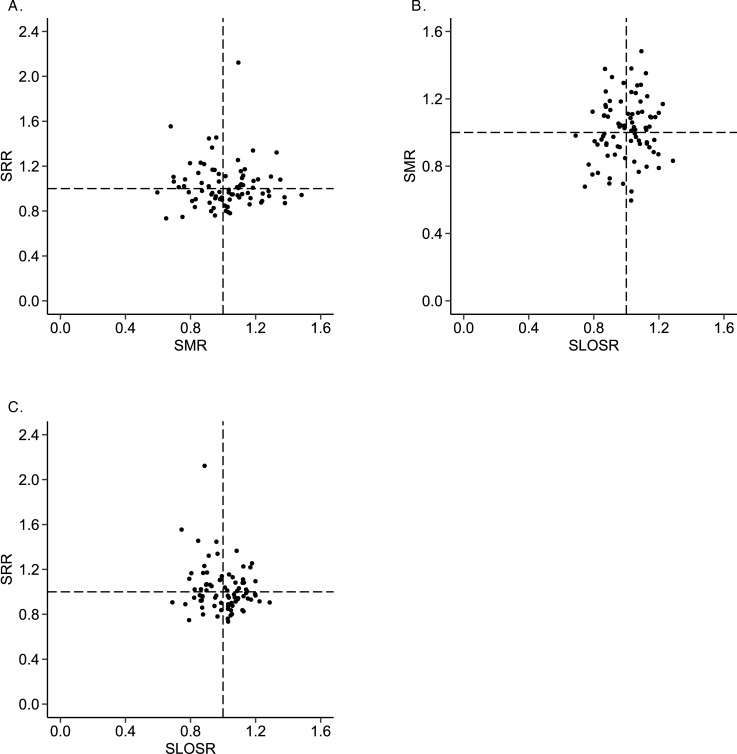
Pairs of quality indicators plotted against each other on ICU level. Fig 2A: standardized in-hospital mortality ratio (SMR) against standardized ICU readmission within 48 hours ratio (SRR). Fig 2B: standardized length of stay ratio (SLOSR) against SMR. Fig 2C: SLOSR against SRR.

**Fig 3:**
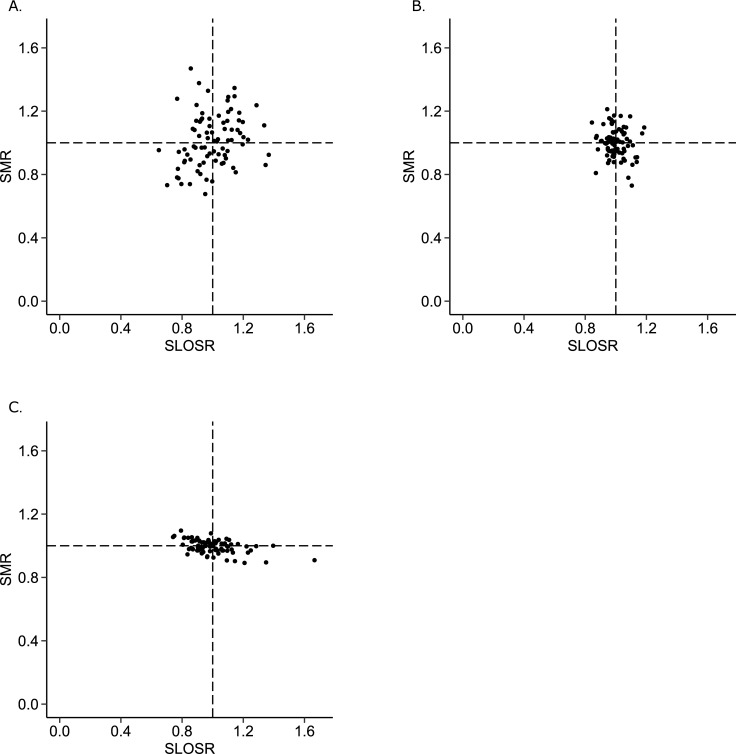
Quality indicators SMR and SLOSR plotted against each other on ICU level for patients grouped based on severity of illness defined using the probability of mortality. Fig 3A: probability of mortality smaller than 0.3. Fig 3B: probability of mortality larger or equal to 0.3 and smaller than 0.7. Fig 3C: probability of mortality larger or equal to 0.7.

Results on the performance of the prediction models are shown in Table D and Figs A- C of S1 File. Fig 2 presents the pairs of SMR, SRR and SLOSR plotted against each other for the entire cohort. Table 2 presents Pearson’s correlation coefficients for the entire cohort as well as for subgroups. No significant associations were identified between SRR and SMR or SRR and SLOSR. This means that ICUs with lower than expected readmission rates did not have lower or higher mortality, or shorter or longer length of stays, than expected. No significant associations were identified at cohort level between SMR and SLOSR. However, a negative significant association (r = -0.49; p<0.001) was found for the high mortality risk subgroup (i.e., recalibrated APACHE IV probability of mortality larger than 0.7), meaning better performance on SMR (lower mortality) was associated with worse performance on SLOSR (longer ICU length of stay). Conversely, a positive association was found for the low mortality risk subgroup (i.e., recalibrated APACHE IV probability of mortality smaller than 0.3). This association was not significant using Pearson’s correlation coefficient (r = 0.25; p = 0.024), but was significant using the Spearman’s rank correlation coefficient (ρ = 0.29; p = 0.009). Fig 3 presents the pairs of SMR and SLOSR for these subgroups based on probability of mortality.

**Table 2:** Pearson’s correlation coefficients with corresponding p-values for the pairs of quality indicators standardized mortality ratio (SMR); standardized readmission ratio (SRR) and standardized ICU length of stay ratio (SLOSR).

	In-hospital SMR and SRR		In-hospital SMR and SLOSR		SLOSR and SRR	
Patient subgroup	Coefficient	p-value^1^	Coefficient	p-value	Coefficient	p-value
All ICU admissions	-0.02	0.877	0.11	0.312	-0.15	0.170
CAP	0.14	0.214	-0.10	0.358	0.10	0.381
Sepsis	0.14	0.199	-0.11	0.330	-0.09	0.429
OHCA	-0.11	0.323	-0.18	0.109	0.02	0.862
*Admission type*						
Medical	-0.05	0.664	0.12	0.264	-0.06	0.614
Urgent Surgery	0.11	0.321	0.04	0.725	-0.12	0.298
Elective surgery	0.17	0.114	0.26	0.018	0.20	0.077
*Probability of mortality*						
<0.3	0.08	0.481	0.25	0.024	-0.14	0.195
≥0.3 and <0.7	-0.11	0.330	-0.07	0.521	-0.05	0.663
≥0.7	-0.22	0.045	-0.49	0.000	-0.07	0.517

^1^P-values were calculated using the Student t-distribution and p-values smaller than 0.01 were viewed as statistically significant.

CAP = Community Acquired Pneumonia; OHCA = Out of Hospital Cardiac Arrest

S3 Table presents Spearman’s rank correlation coefficients for the entire cohort as well as for subgroups. These results are compatible with the results from the primary analysis.

## Discussion

In this study we examined the association between SMR, SLOSR and SRR as individual outcome quality indicators for benchmarking ICU quality of care. We did not find significant correlations between SRR and SMR or SRR and SLOSR. This means that ICUs with lower than expected readmission rates did not have lower or higher mortality, or shorter or longer length of stays, than expected. We also did not find a significant correlation between SMR and SLOSR for the general ICU population. However, subgroup analyses based on probability of mortality showed associations in opposite directions.

Among the high-risk patient population the association between SMR and SLOSR was negative, i.e. better performance on SMR (lower mortality) was associated with worse performance on SLOSR (longer ICU length of stay). One of the explanations could be the process of end-of-life decisions, which might differ between ICUs. Among the low-risk patient population there was a non-significant positive association between SMR and SLOSR. One explanation might be that problems in the care process resulting in more complications during ICU stay may for example cause higher mortality and longer ICU length of stay.

Although results from subgroups should interpreted cautiously, such different directions in the associations between SMR and SLOSR depending on mortality-risk could be plausible. High quality of care could result in both lower mortality and earlier ICU discharge, explaining the positive association in low-risk patients. However, patients who die in a specific ICU may die later in another ICU. Thus, if mortality is high, this may paradoxically decrease the mean ICU length of stay, explaining the negative association between SMR and SLOSR in high-risk populations.

On patient level several studies found that patients that survived their initial ICU stay and are readmitted to the ICU showed higher mortality rates and longer length of stay [13, 16, 36] compared to patients not readmitted to the ICU. However, studies using quality indicators after correction for case-mix reported similar results as ours. A recent study on Dutch hospitals found that hospitals with many patients with long length of stay also had higher in-hospital mortality rates, but they found no correlation for readmissions [37]. However, for Dutch ICU patients in-hospital mortality predictions seems to be more reliable using clinical data compared to administrative data [38]. Two earlier studies found no association between quality indicators for ICU patients after case-mix correction [13, 36]. For all hospitalized patients one study found no correlation between SMR and readmission rate, but length of stay was positively correlated with SMR [39]. These results may differ since they are not specific for ICU patients, hospital length of stay was used and no-case mix correction was applied for length of stay. Two other studies on hospitalized patients also found that a decrease in length of stay may not necessarily lead to increases in hospital readmission rates [14] and that hospitals with different levels of performance on mortality showed similar results on hospital readmission rates [40].

Interestingly, in our pre-defined subgroup analyses, an association between SMR and SLOSR was identified. In patients with high mortality risk, a high SMR was associated with a shorter case-mix corrected ICU length of stay. In contrast a non-significant opposite association was found in low-risk patients. Although results from subgroups should interpreted cautiously, such different directions in the associations between SMR and SLOSR depending on mortality-risk could be plausible. High quality of care could result in both lower mortality and earlier ICU discharge, explaining the positive association in low-risk patients. However, patients who die in a specific ICU may die later in another ICU. Thus, if mortality is high, this may paradoxically decrease the mean ICU length of stay, explaining the negative association between SMR and SLOSR in high-risk populations.

In this study, we focused on three quality indicators related to the outcomes of ICU patients. Within the ICU domain many other quality indicators, such as structure and process indicators are also used [4, 41]. For example, within the NICE registry new actionable quality indicators for pain management, blood use, antibiotics and mechanical ventilation have been recently developed [42]. Their aim is to provide tools to directly improve specific care processes. This is in contrast to the three quality indicators examined in this study, which provide a more general view on overall quality of ICU care. For the total cohort of ICU patients the risk of bias in our study was low and the generalizability was high, since many patient level data were available and because all Dutch ICUs participated in this study. Although we do not know the level of residual confounding, we believe that it is small because case-mix correction was applied using a well-established mortality prediction model (APACHE IV) and the models for predicting readmissions and length of stay were developed on our data using a broad set of predictive variables, all measured during the first 24 hours of ICU admission.

ICU discharge decisions often do not only depend on a patient's recovery, but on organizational circumstances such as availability of beds on the general ward and the need to free up ICU beds for other patients. Nevertheless, we have deliberately chosen not to include ICU and hospital level covariates in our prediction model for ICU length of stay, since our study has been performed in the context of benchmarking where one does not want to adjust for but detect organizational differences. Furthermore, in a previous study, we found that after correcting for patient characteristics including ICU characteristics did not significantly improve ICU LoS predictions [43].

Calibration of all three models was satisfactory, but we did find low values of the scaled Brier score and C-statistic for readmission predictions, Table C of S1 File. This indicates that there was variation in readmission rates that could not be explained by case-mix factors; we do not believe that it has influenced our findings. Our model to predict the probability of being readmitted within the first 48 hours after ICU discharge relies on patient data reflecting severity of illness available from the first 24 hours of ICU admission [24]. It may be better to take severity of illness upon ICU discharge into account [16, 17], but these data are not available in our registry.

A limitation of our approach is that some quality indicator values were estimated from small subgroups. For subgroups of ICU patients with a small number of admissions, estimators of quality indicator scores for these small ICUs would be less reliable than for ICUs with a larger number of admissions, which may have resulted in unstable estimates (i.e. of small ICUs) towards 1 [27, 28]. As a solution, for each subgroup we decided to apply reliability adjustment to shrink the observed indicator values to the overall average. A sensitivity analysis using the more robust rank correlation coefficient and analysis without adjustment for reliability showed similar results as the main analysis. However, for benchmarking purposes we think that it is not convenient to present results on ICU-level based on very few patients.

Our criteria for patient inclusion lead to several limitations. Firstly, the exclusion of patients that were transferred to another ICU or are discharged from the hospital in order to get palliative care elsewhere may have biased the SMR values. These patients are known to have higher mortality rates [44]. Using long term mortality as outcome measure compared to in-hospital mortality may be useful [45–47]. We found that the number of transferals in the Dutch ICU system was 1.2% after applying all other patient exclusion criteria. Secondary analyses showed that using SMR based on three months mortality instead of in-hospital mortality did not change the results regarding associations between SMR, SRR and SLOSR (results not shown). Secondly, no information was available regarding readmission to the ICU for patients discharged from the hospital, which may have biased our estimates of SRR values. Thirdly, death or hospital discharge within 48 hours could be competing risks with readmission to the ICU and was not taken into account. Patients who die or are discharged from the hospital within 48 hours after ICU discharge may have a lower probability to be readmitted within 48 hours after ICU discharge compared to patients who are not discharged from the hospital within 48 hours after ICU discharge.

## Conclusion

We identified no significant association between quality indicators for in-hospital mortality, readmission to the ICU within 48 hours after ICU discharge and ICU length of stay at ICU population level. Differential associations were found between performance on mortality and length of stay within different risk strata. We recommend users of quality information for benchmarking purposes to take these three outcome indicators into account when judging or monitoring ICU quality of care as they capture different aspects of ICU performance. Furthermore, we suggest that users of quality information also receive quality data for patient subgroups, especially low-risk and high-risk patients groups.

## Supporting information

S1 Table Definitions of patients subgroups included in the study. (PDF)

S2 Table Number of included ICUs and number of included admissions for each subgroup and quality indicator. Overall values are shown accompanied with minimum and maximum ranges over the ICUs.
(PDF)

S3 Table Spearman rank correlation coefficients with corresponding p-values for the pairs of quality indicators standardized mortality ratio (SMR); standardized readmission ratio (SRR) and standardized ICU length of stay ratio (SLOSR). (PDF)

S1 File Results of recalibration and regression analysis to calculate predicted outcome. (PDF)
