# Supplementary material for: The association between outcome-based quality indicators for intensive care units
Source: PLoS One. 2018 Jun 13;13(6):e0198522. doi: 10.1371/journal.pone.0198522 (PMC5999279; doi:10.1371/journal.pone.0198522)
Supplement: S1 Table — (PDF) [file pone.0198522.s001.pdf]

Table S1. Definitions of patients subgroups included in the study

**Subgroups of ICU admissions**	**Definition in this study**
Community acquired pneumonia (CAP)	APACHE IV admission diagnose pneumonia: aspiration; bacterial; fungal; parasitic; viral; other. Hospital admission prior to ICU admission less than 48 hours.
Sepsis	APACHE IV admission diagnose sepsis by infection site: cutaneous/soft tissue; gastrointestinal; Gynecologic; pulmonary; renal/urinary tract; other location; or unknown location
Out of Hospital Cardiac Arrest (OHCA)	Cardio pulmonary resuscitation in the 24 hours before ICU admission or APACHE IV diagnose cardiac arrest (with or without respiratory arrest; for respiratory arrest see Respiratory System).<br><br>Patient admitted directly from home or from an emergency department in their own hospital or other hospital.
Admission type	
Medical	All admissions not admitted directly from operating or recovery room.
Urgent Surgery	Immediate surgery where resuscitation, stabilization and physiological optimization occur simultaneously or immediately prior to surgery.
Elective surgery	Surgery at a time that both patient and surgeon schedule or early surgery scheduled within 24 hours after OK indication.
Probability of mortality	
<0.3	Admissions with recalibrated APACHE IV probability smaller than 0.3.
≥0.3 and <0.7	Admissions with recalibrated APACHE IV probability larger or equal than 0.3 and smaller than 0.7.
≥0.7	Admissions with recalibrated APACHE IV probability larger or equal than 0.7.
