# Supplementary material for: The association between outcome-based quality indicators for intensive care units
Source: PLoS One. 2018 Jun 13;13(6):e0198522. doi: 10.1371/journal.pone.0198522 (PMC5999279; doi:10.1371/journal.pone.0198522)
Supplement: S2 Table — Overall values are shown accompanied with minimum and maximum ranges over the ICUs. (PDF) [file pone.0198522.s002.pdf]

Table S2.Number of included ICUs and number of included admissions for each subgroup and quality indicator. Overall values are shown accompanied with minimum and maximum ranges over the ICUs.

Patient subgroup	Number of ICUs	Number of admission (min to max) for ICU length of stay and in-hospital mortality	Number of admission (min to max) for readmission to the ICU
All ICU admissions	83	59,809 (205 to 2,282)	48,496 (148 to 1,974)
CAP	81	1,286 (1 to 113)	876 (1 to 59)
Sepsis	83	2,843 (2 to 107)	2,135 (2 to 90)
OHCA	78	2,150 (1 to 103)	1,110 (1 to 52)
Admission type			
Medical	83	34,096 (49 to 1,137)	24,881 (36 to 838)
Urgent surgery	83	8,000 (14 to 357)	6,908 (13 to 299)
Elective surgery	83	17,713 (12 to 918)	16,707 (12 to 911)
Probability of mortality			
<0.3	83	46,971 (160 to 1,877)	40,858 (123 to 1,716)
≥0.3 and <0.7	83	8,671 (11 to 288)	6,137 (6 to 219)
≥0.7	83	4,167 (1 to 166)	1,501 (1 to 78)
