# Supplementary material for: The association between outcome-based quality indicators for intensive care units
Source: PLoS One. 2018 Jun 13;13(6):e0198522. doi: 10.1371/journal.pone.0198522 (PMC5999279; doi:10.1371/journal.pone.0198522)
Supplement: S3 Table — (PDF) [file pone.0198522.s003.pdf]

Table S3. Spearman rank correlation coefficients with corresponding p-values for the pairs of quality indicators standardized mortality ratio (SMR); standardized readmission ratio (SRR) and standardized ICU length of stay ratio (SLOSR).

	In-hospital SMR and SRR		In-hospital SMR and SLOSR		SLOSR and SRR	
Patient subgroup	Coefficient	p-value[1]	Coefficient	p-value	Coefficient	p-value
All ICU admissions	-0.01	0.925	0.09	0.407	-0.05	0.671
CAP	0.07	0.560	-0.13	0.238	-0.09	0.449
Sepsis	0.19	0.086	-0.09	0.402	-0.11	0.317
OHCA	0.00	0.994	-0.17	0.145	0.03	0.781
Admission type						
Medical	-0.01	0.937	0.12	0.271	0.03	0.799
Urgent Surgery	0.10	0.375	0.03	0.818	-0.07	0.556
Elective surgery	0.19	0.087	0.31	0.004	0.20	0.075
Probability of mortality						
<0.3	0.08	0.500	0.29	0.009	-0.11	0.333
≥0.3 and <0.7	-0.15	0.171	-0.15	0.190	-0.01	0.951
≥0.7	-0.26	0.016	-0.41	0.000	-0.09	0.424

[1]P-values were calculated using Spearman's rank correlation test and p-values less than 0.01 were viewed as statistically significant.
