# Supplementary material for: The association between outcome-based quality indicators for intensive care units
Source: PLoS One. 2018 Jun 13;13(6):e0198522. doi: 10.1371/journal.pone.0198522 (PMC5999279; doi:10.1371/journal.pone.0198522)

Regression model in R for in-hospital mortality prediction

```
formula_mortality <- [in-hospital death]~Logit([APACHE IV probability])+
                                        [admission type] *rcs([APACHE III score])
fit<-glm(formula=formula_mortality,data=dataset,family=binomial())
```

Table A. Recalibration of the APACHE IV probability of mortality by logistic regression and in-hospital mortality as dependent variable.

**Patient-characteristics**	**Coefficient (Wald-type confidence interval)**	**p-value (Chi-squared test)**
Intercept	-2.22	0.015
Logit APACHE IV probability of mortality	0.92	<0.001
Admission type (reference elective surgery)		
Medical	1.00	0.339
Urgent	-2.09	0.319
Spline APACHE III score (reference category, elective surgery)		
First spline coefficient	0.02	0.431
Second spline coefficient	0.01	0.976
Third spline coefficient	0.04	0.948
Fourth spline coefficient	-0.19	0.770
Medical admission type x spline APACHE III score		
First spline coefficient	-0.01	0.653
Second spline coefficient	0.11	0.662
Third spline coefficient	-0.59	0.448
Fourth spline coefficient	0.83	0.242
Urgent surgery admission type x spline APACHE III score		
First spline coefficient	0.07	0.258
Second spline coefficient	-0.60	0.174
Third spline coefficient	1.79	0.160
Fourth spline coefficient	-1.51	0.158

Regression equation
Logit(expected mortality)=-2.22
+0.92*[logit APACHE IV probability of mortality]
+1.00*[Medical admission]
-2.09*[Urgent surgery admission]
+0.02*[APACHE III score]
+6.93E-07*([APACHE III score]-20)^3
+4.62E-06*([APACHE III score]-39)^3
-1.93E-05*([APACHE III score]-53)^3
+1.75E-05*([APACHE III score]-71)^3
-3.49E-06*([APACHE III score]-118)^3
-0.01*[APACHE III score]*[Medical admission]
+1.15E-5*([APACHE III score]-20)^3*[Medical admission]
-6.13E-5*([APACHE III score]-39)^3*[Medical admission]
+8.64E-5*([APACHE III score]-53)^3*[Medical admission]
-4.05E-5*([APACHE III score]-71)^3*[Medical admission]
+3.84E-6*([APACHE III score]-118)^3*[Medical admission]
+0.07*[APACHE III score]*[Urgent surgery admission]
-6.23E-5*([APACHE III score]-20)^3*[Urgent surgery admission]
+1.86E-4*([APACHE III score]-39)^3*[Urgent surgery admission]
-1.57E-4*([APACHE III score]-53)^3*[Urgent surgery admission]
+3.48E-5*([APACHE III score]-71)^3*[Urgent surgery admission]
-1.22E-6*([APACHE III score]-118)^3*[Urgent surgery admission]

Regression model in R for prediction of readmission to the ICU

```
formula_readmission<- [readmitted to the ICU within 48 hours] ~
                          [Logit([APACHE IV probability])]+[age] +
                          [planned admission]+[medical admission]+
                          [urgent surgery admission]+[vasoactive drug]+
                          [confirmed infection]+[copd]+[cva]+
                          [mechanical ventilation first 24h]+
                          [cardiovascular insufficiency]+[neoplasm]+
                          [chronic renal insufficiency]+[cirrhosis]+
                          [chronic dialysis]+[immunologic insufficiency]+
                          [chronic respiratory insufficiency]+
                          [hematologic malignancy ]+[ acute renal failure]+
                          [gastro intestinal bleeding]
 fit <- glm(formula <- formula_readmission, data = dataset, family = binomial())
```

Table B. Regression coefficients of logistic regression with patient readmitted to the ICU during the same hospital admission period as dependent variable.

	**Coefficient (Wald-type confidence interval)**	**p-value (Chi-squared test)**
Intercept	-3.56 (-4.01 to -3.11)	<0.001
Logit APACHE IV probability of mortality	0.13 (0.08 to 0.18)	<0.001
Age	0.00 (0.00 to 0.01)	0.689
Planned admission	-0.11 (-0.33 to 0.1)	0.311
Admission type (reference elective surgery)		
Medical	-0.18 (-0.4 to 0.04)	0.102
Urgent surgery	-0.03 (-0.27 to 0.2)	0.774
Confirmed infection (yes)	0.16 (0.01 to 0.31)	0.041
Mechanical ventilation first 24 hours (yes)	0.36 (0.23 to 0.5)	<0.001
Vasoactive drug use first 24-hours of admission (yes)	0.05 (-0.09 to 0.18)	0.484
Chronic diagnoses (yes)		
Cardiovascular insufficiency	-0.18 (-0.52 to 0.15)	0.283
Chronic Obstructive Pulmonary Disease (COPD)	0.15 (-0.01 to 0.31)	0.068
Chronic renal insufficiency	0.00 (-0.27 to 0.27)	0.992
Chronic dialysis	0.22 (-0.28 to 0.72)	0.396
Chronic resperatoir insufficiency	0.00 (-0.28 to 0.27)	0.988
Cirrhosis	0.09 (-0.36 to 0.54)	0.689
Hematologic malignity	0.26 (-0.12 to 0.63)	0.187
Immunologic insufficiency	0.13 (-0.06 to 0.33)	0.187
Neoplasm	-0.3 (-0.57 to -0.02)	0.033
Acute diagnoses (yes)		
Acute renal failure	-0.13 (-0.34 to 0.09)	0.255
Cardio vascular accident (CVA)	-0.14 (-0.42 to 0.15)	0.350
gastro-intestinal bleeding	0.55 (0.24 to 0.87)	0.001

Regression model in R for prediction of ICU length of stay

As independent variables we included an initial group of patient characteristics and performed a stepwise backwards selection procedure, excluding the variable with the highest p-value at each step until all variables remaining in the model had p-value less than 0.1.

```
splineaps <- rcs([APACHE IV aps score],parms = 3)
splineage <- rcs([age],parms = 3)
formula_LoS <- [ICU length of stay] ~ [gender]+[splineaps]+[splineage]+
                    [admission type]+[mechanical ventilation first 24h]+
                    [confirmed infection]+[vasoactive drug]+[diabetes]+[copd]+
                    [Lowest GCS first 24h]+[immunologic insufficiency]+
                    [APACHE IV admission diagnose]+[cpr]+[dysrhythmia]+
                    [gastro intestinal bleeding] +[cva]+[intracranial mass]+
                    [acute renal failure]+[ chronic renal insufficiency]+
                    [chronic dialysis]+[chronic respiratory insufficiency]+
                    [cardio vascular insufficiency]+[hematologic malignancy]
model.caseMix <- glm(formula <- formula_LoS,data=dataset,family = gaussian(link='log'))
model.caseMix1 <- step(model.caseMix)
```

Table C. Regression coefficients of ordinary least square regression with intensive care unit length of stay as outcome measure.

Patient-characteristics	Coefficient (Wald-type confidence interval)	p-value (Chi-squared test)	Patient-characteristics	Coefficient (Wald-type confidence interval)	p-value (Chi-squared test)
Intercept	-1.41 (-1.56 to -1.25)	<0.001	*Acute diagnoses (yes)*		
Gender: male	0.07 (0.05 to 0.1)	<0.001	Acute renal failure	0.21 (0.18 to 0.24)	<0.001
Admission type			Cardiopulmonary reanimation (CPR)	-0.08 (-0.13 to -0.03)	0.003
Urgent surgery	-0.46 (-1.12 to 0.2)	0.169	Dysrhytmia	0.04 (0.01 to 0.07)	0.023
Elective surgery	-0.71 (-1.37 to -0.06)	0.033	Intracranial mass effect	0.21 (0.15 to 0.27)	<0.001
Age			*APACHE IV admission diagnose (head categories)*		
First coefficient of spline for age	0.01 (0.01 to 0.01)	<0.001	Gastro-intestinal non-operative	0.03 (-0.03 to 0.1)	0.318
Second coefficient of spline for age	-0.01 (-0.02 to -0.01)	<0.001	Genito-uritary non-operative	-0.21 (-0.33 to -0.09)	<0.001
APACHE IV physiology score (APS)			Hematology non-operative and operative	0.21 (0.09 to 0.34)	<0.001
First coefficient of spline for APS	0.04 (0.04 to 0.04)	<0.001	Metabolic non-operative	-0.18 (-0.32 to -0.03)	0.015
Second coefficient of spline for APS	-0.04 (-0.05 to -0.04)	<0.001	Musculoskeletal/skin non-operative	0.13 (-0.07 to 0.34)	0.207
Confirmed infection (yes)	0.31 (0.28 to 0.34)	<0.001	Neurological non-operative	0 (-0.06 to 0.06)	0.977
Mechanical ventilation first 24 hours (yes)	0.55 (0.52 to 0.59)	<0.001	Respiratory non-operative	0.23 (0.19 to 0.27)	<0.001
Vasoactive drug use first 24-hours of admission (yes)	0.29 (0.26 to 0.32)	<0.001	Transplant operative	0.01 (-0.6 to 0.62)	0.966
Lowest GCS the first 24-hours of ICU admission	0.02 (0.01 to 0.02)	<0.001	Trauma non-operative	0.35 (0.27 to 0.42)	<0.001
Chronic diagnoses (yes)			Cardiovasculair operative	0.58 (-0.08 to 1.24)	0.084
Chronic Obstructive Pulmonary Disease (COPD)	-0.09 (-0.12 to -0.06)	<0.001	Gastro-intestinal operative	0.43 (-0.23 to 1.08)	0.204
Chronic renal insufficiency	-0.06 (-0.11 to -0.01)	<0.001	Genito-uritary operative	0.1 (-0.58 to 0.78)	0.772
Chronic dialysis	-0.3 (-0.43 to -0.17)	<0.001	Metabolic operative	0.22 (-0.69 to 1.13)	0.637
Chronic resperatoir insufficiency	0.04 (0 to 0.09)	0.054	Musculoskeletal/skin operative	0.08 (-0.61 to 0.76)	0.827
Cirrhosis	-0.29 (-0.38 to -0.19)	<0.001	Neurological operative	0.6 (-0.06 to 1.26)	0.076
Diabetes	-0.04 (-0.07 to -0.01)	0.007	Respiratory operative	0.48 (-0.18 to 1.15)	0.155
Hematologic malignity	-0.05 (-0.12 to 0.01)	0.113	Trauma operative	0.73 (0.07 to 1.39)	0.031
Immunologic insufficiency	0.1 (0.06 to 0.13)	<0.001			
Neoplasm	-0.32 (-0.38 to -0.25)	<0.001			

Variables excluded by stepwise regression: hematologic malignity; resperatroy insufficiency; cardio vascular insufficiency; dysrhytmia

Regression formula for spline variables for APACHE III score:

4.17E-2*[APACHE III score]-1.02E-5*([APACHE III score]-19)^3+1.50E-5*([APACHE III score]-40)^3-4.77E-6*([APACHE III score]-85)^3

Regression formula for spline variables for age: 6.76E-3*[age]-7.66E-6*([age]-39)^3+2.06E-5*([age]-66)^3-1.29E-5*([age]-82)^3

Performance of the case-mix adjustment models

Table D. Performance results for the predicted probability of mortality; predicted probability of readmission within 48 hours; and ICU length of stay predictions on patient.

All ICU admissions[1]	R^2 on patient level[1]	Scaled Brier score[2]	C-statistics[2]	Calibration curve[3]	
				α	β
Predicted probability of mortality		0.33	0.89	0.00 (0.00 to 0.01)	0.99 (0.97 to 1.01)
Predicted probability of readmission		0.00	0.61	0.00 (0.00 to 0.01)	0.95 (0.79 to 1.12)
Predicted ICU length of stay	0.16			0.06 (-0.01 to 0.14)	0.99 (0.97 to 1.01)

[1]Squrared Pearson's correlation coefficient (R^2) was used for continuous outcomes.
[2]For the dichotomous outcome measures the Brier skill score and C-statistics were used.
[3]Calibration is based on 50 subgroups of 2% percentiles of predicted outcome.

Fig A. Calibration plot of predicted in-hospital mortality against observed in-hospital mortality, based on 2% percentiles of predicted probability of mortality.

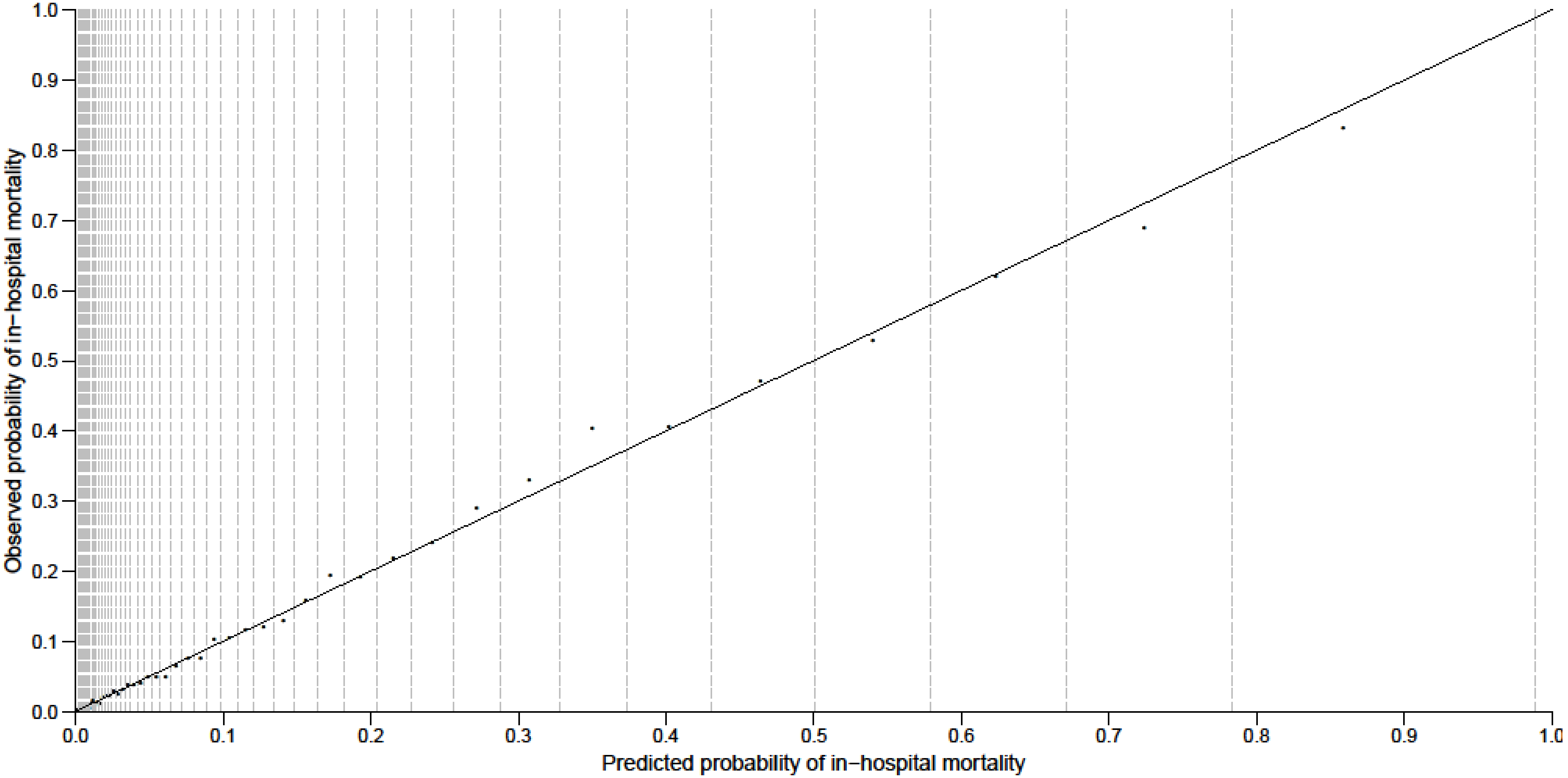

Fig B. Calibration plot of predicted probability of readmission to the ICU against observed probability of readmission to the ICU within 48 hours of ICU discharge, based on 2% percentiles of predicted probability of readmission to the ICU.

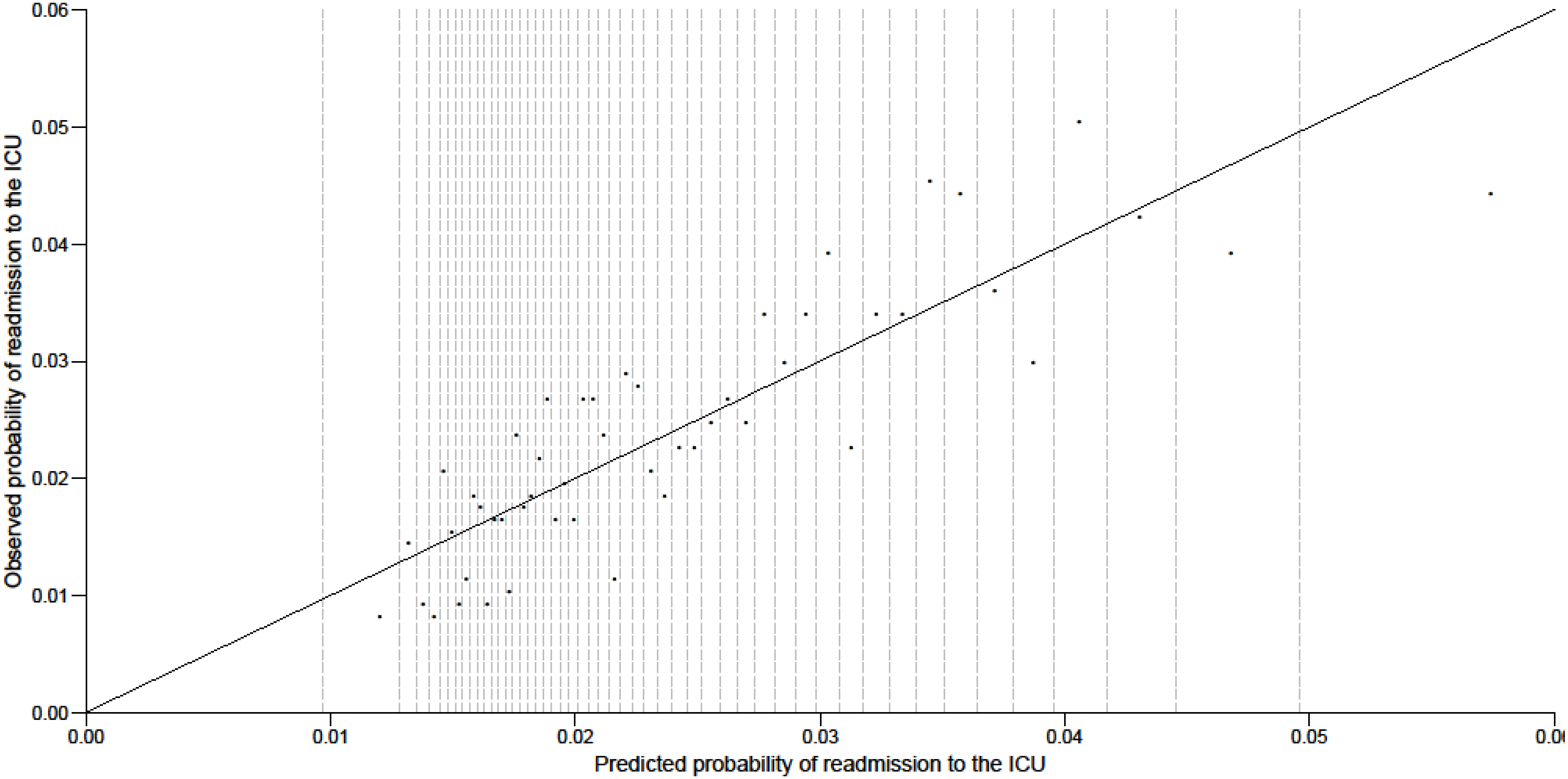

Fig C. Calibration plot of mean predicted ICU length of stay against mean observed ICU length of stay, based on 2% percentiles of predicted ICU length of stay.

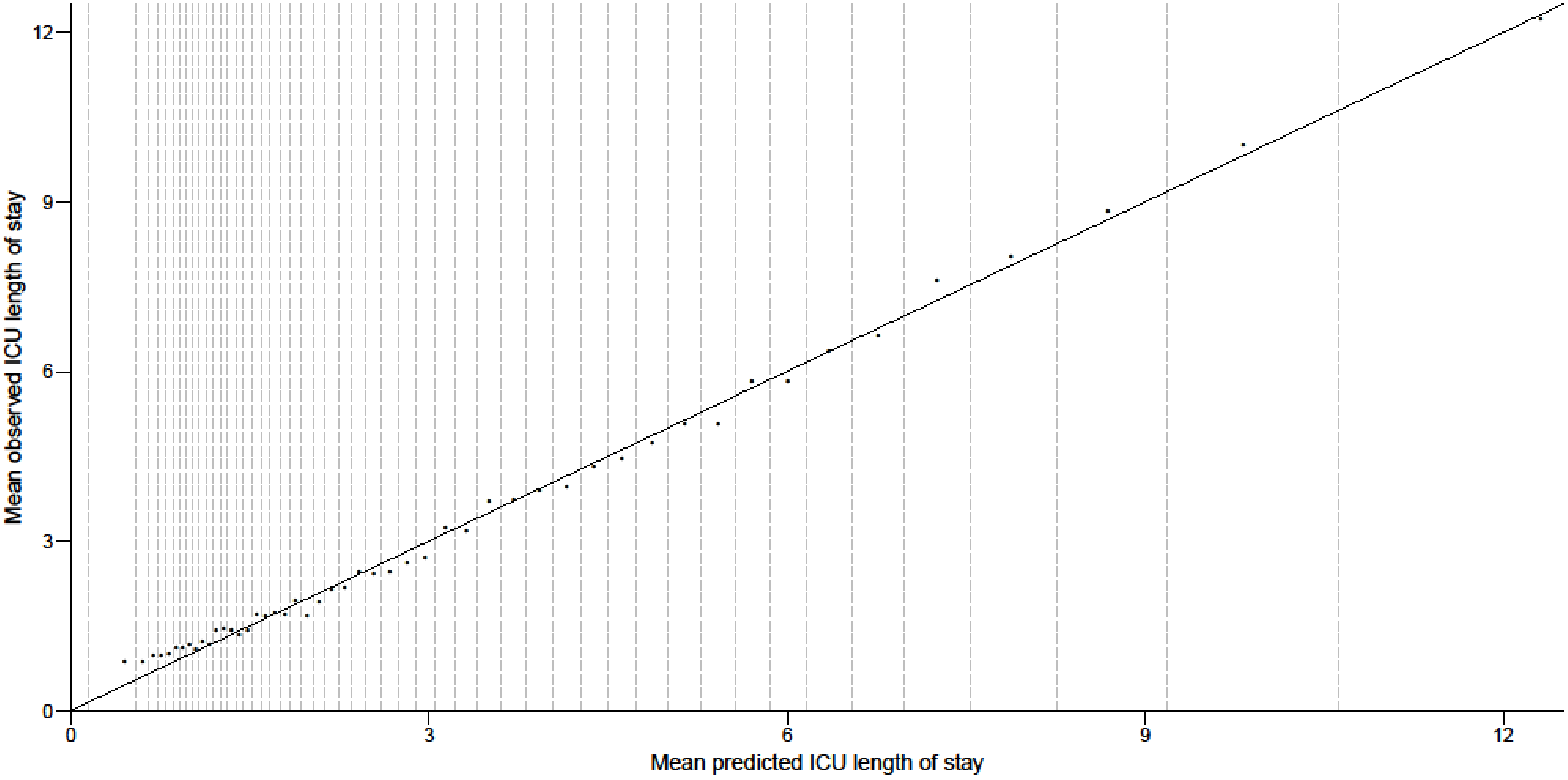

Supplement: S1 File — (PDF) [file pone.0198522.s004.pdf]
